# Non-visible disease, the hidden disruptive experiences of chronic illness in adversity

**DOI:** 10.1080/17482631.2020.1857579

**Published:** 2020-12-07

**Authors:** Sara Estecha Querol, Pam Clarke, Elisabeth Lilian Pia Sattler, Jason C. G. Halford, Mark Gabbay

**Affiliations:** a Institute of Population Health, University of Liverpool, Liverpool, UK; b Academic Unit of Primary Care, Warwick Medical School, University of Warwick, Coventry, UK; c Department of Foods and Nutrition and Department of Clinical and Administrative Pharmacy, University of Georgia, Athens, GA, USA; d School of Psychology, University of Leeds, Leeds, UK

**Keywords:** Chronic illness experience, cardiovascular disease, biographical disruption, health inequalities, disruptive experience, adversity

## Abstract

**Objective**: This study’s principal aim was to describe the lived reality for people with cardiovascular disease (CVD) and other chronic health conditions, who live in economically deprived neighbourhoods in a city in North West England.

**Methodology**: This is a qualitative, exploratory study based on in-depth, semi-structured interviews with participants experiencing compromised cardiovascular health, conducted in August 2017. The study sample comprised 14 adults (3 females) aged 54 to 76. Thematic analysis was used for data analysis, and the biographical disruption concept was used as theoretical reference to explore the results. Wider health inequalities literature supplemented the individual experiences of chronic illness.

**Results**: Four main themes were developed from the data: (1) chronic illness as a disruptive experience; (2) struggling for money; (3) lifestyle and health risks; and (4) reflections on current inequalities. The varied nature of participants’ narratives about their chronic illness indicated that the experience of biographical disruption depends on the wider socioeconomic and cultural factors of the individual.

**Discussion**: This study suggests that biographical disruption theory combined with health inequalities contexts highlights the role of hidden suffering and enhances the understanding of chronic illness experiences and thus informs clinical management, service and public health planning.

## Introduction

Bury (1982) established the three key elements of the biographical disruption theory. These are: an explanation of how illness interrupts the future plans of an individual, including taken-for-granted features; a rethinking of the personal biographies and self-concept; and an attempt to normalize the individual´s disrupted life by using material and social resources. Yet, the concept of biographical disruption was later reviewed and extended (S. Williams, 2000a). Chronic illness may be biographically anticipated or considered to be inevitable in old age rather than a disruption (Pound et al., 1998). Furthermore chronic illness may be experienced as biographical continuity or flow as a part of an ongoing life story (Faircloth et al., 2004). Illness could act as confirmation or reinforcement of a pre-existing chronic illness experience (Carricaburu & Pierret, 1995). Chronic illness could also be experienced as a “death sentence”, where life is perceived as already over (biographical abruption) (Locock et al., 2009), while biographical reinstatement reflects normalization of symptoms and integration of illness experience into normal life (Sanderson et al., 2011). Lastly, the biographical work concept has been used to explain symptomless chronic conditions, in which the illness does not provide bodily signs of sickness and disability (Felde, 2011).

However, biographical disruption is not sufficient to explain the diversity of experiences of chronic illness, as its focus on the illness may mask and reduce the impact of the wider socioeconomic and cultural factors on the chronic condition of the individual (Lawton, 2003; S. Williams, 2000a). It is essential to frame the experiences of chronic illnesses in their broad context. In this study, we expanded this original focus to include a more detailed look at the economic, lifestyle, and environmental factors that impinge on the degree of biographical disruption. There is a need to supplement or go beyond the micro context of the individual framed within wider health inequalities literature.

Health inequalities are defined as differences in health status or in the distribution of health determinants between different population groups (World Health Organization, 2008), and are considered “avoidable and unfair” (Whitehead, 1992). In the English context, the North West Coast is among the most disadvantaged regions, facing high rates of health inequalities (Whitehead, 2014; Wood et al., 2006) and deprivation (Department for Communities and Local Government, 2015). Higher unemployment rates, lower wages, higher levels of chronic illness and disability, and poorer working and living environments are some of the socioeconomic causes of health inequalities that are affecting North West England (National Institute for Health Research, 2017).

We conducted this study in an economically deprived area from the North West England in order to develop greater understanding of the impact of economic, lifestyle, and environmental determinants, within the wider socioeconomic context, on people’s experience of chronic ill-health. Therefore, our principal aim was to understand the lived reality of people managing chronic illnesses in economically deprived neighbourhoods in a city in North West England. Participants’ behaviours and reflections were framed by their socioeconomic context: individuals living in a low-income area of a developed country. The biographical disruption framework was used to understand these individual experiences of chronic illness.

## Methodology

### Recruitment

We purposively sampled participants for age and gender (Jupp, 2006) in a neighbourhood which is amongst the 20% most deprived neighbourhoods in the country, according the IMD 2015 (Department for Communities and Local Government, 2015). Snowballing was used as a sampling strategy facilitating access and identifying potential participants. Researchers’ and participants’ networking helped to recruit interested and eligible individuals. Two authors (SEQ and PC) mapped the area and established contact with public organizations in the area by asking key people to forward information about the study (participant information sheet and a flyer) to any eligible participants. We also used word of mouth plus advertisements in public spaces. Recruitment was challenging due to the nature of the group: low income individuals tend not to get involved in research (Braun & Clarke, 2013), as well as the sensitive nature of the topic. However, fourteen interviews were conducted of which twelve took place in the Stroke Association venue and the remaining two in the participant’s home. We identified that people were more likely to take part in the study if they were a member of a well-established group (e.g., Stroke Association) rather than unaffiliated individuals that we met in other places such as the food bank, federations, local shops or libraries. The researchers (SEQ and PC) were introduced to the members of the Stroke Association by the managers, leading to an atmosphere of trust. In contrast, individual people might not have called us to set up an interview, or decided against the interview after an initial conversation because they might not have been sure of the study or the researchers.

### Methods of data collection

Conversational, topic-guided, in-depth, semi-structured qualitative interviews were conducted with participants experiencing compromised cardiovascular health. The topic guide covered three key elements: 1) chronic illness and its management; 2) dietary choices and food bank utilization; and 3) trade-off decision making regarding basic needs, including food, medications, and other necessities. Participants gave informed consent for the interviews, which were audio recorded. A short questionnaire on general demographic information was also completed.

Ethics approval was obtained (reference number 1815 University of Liverpool). All data were pseudo-anonymized, with removal of identifiers.

### Participant characteristics

Participants were recruited from a deprived North West England inner-city neighbourhood. All participants were living in a low-income area, and they self-reported at least one major risk factor for or the existence of cardiovascular disease (CVD).

Fourteen qualitative interviews were conducted, at which point the research team agreed that thematic saturation had been achieved. Table I shows the characteristics of the study participants. The sample was comprised of 14 people (3 females) aged betwen 54 and 76 (mean = 62.5). Eleven participants had suffered a stroke. Many had other chronic health conditions, such as obesity, depression, epilepsy, and diabetes. Self-identified ethnicity of all participants was white British except one mixed ethnic group. None of the participants were working, and all were receiving welfare/sickness benefits. Two of the participants reported using food banks. We used the Index of Multiple Deprivation (IMD) 2015 (Department for Communities and Local Government, 2015) in order, where possible, to identify the living conditions of the participants and to understand how these conditions might have shaped participants’ narratives. The IMD is an official measure of relative deprivation for small areas in England. It ranks every small area in England called Lower-layer Super Output Areas (LSOA) from 1 (most deprived area) to 32,844 (least deprived area). The IMD combines information using appropriate weights from seven domains (Income Deprivation; Employment Deprivation; Health Deprivation and Disability; Education, Skills and Training Deprivation; Crime; Barriers to Housing and Services; and Living Environment Deprivation).

**Table I.** Characteristics of the sample

Pseudonym	Age	Marital status	Employment status	Monthly household income	IMD 2015a	Self-reported chronic illness(es)	Self-reported CVD risk factors
Terry	56	Divorced	Unfit to work	More than £800	Not available	Stroke, obesity	Family history of CVD, smoking, poor diet, high blood cholesterol levels, obesity, physical inactivity
Diana	76	Divorced	Retired	More than £800	20 per cent most deprived neighbourhood	Stroke, epilepsy, arthritis, diabetes, obesity, liver problems	Heavy smoker in the past, poor diet, high blood pressure, high blood cholesterol levels, diabetes, obesity
Stuart	55	Single	Unfit to work	Less than £400	20 per cent most deprived neighbourhood	Arthritis, depression, anxiety, obesity	Family history of CVD, poor diet, high blood pressure, obesity, physical inactivity
Derek	68	Married/partner	Retired	More than £800	30 per cent most deprived neighbourhood	Angina, stroke, arteriosclerosis, cervical spondylosis.	Family history of CVD, heavy smoker in the past, high blood pressure, high blood cholesterol levels
Robert	55	Single	Unfit to work	More than £800	20 per cent most deprived neighbourhood	Stroke, peripheral arterial disease, diabetes, obesity, vision problems	Heavy smoker, poor diet, high blood pressure, high blood cholesterol levels, diabetes, obesity
James	55	Single	Unemployed with benefits	Between £400—£800	50 per cent most deprived neighbourhood	Stroke, depression	Family history of CVD, poor diet
Clare	58	Single	Unfit to work	Less than £400	40 per cent most deprived neighbourhood	Brain haemorrhage	High blood cholesterol levels
Louis	75	Married/partner	Retired	Does not know	40 per cent least deprived neighbourhood	Stroke, diabetes	Smoker in the past, high blood pressure, diabetes
Jacob	53	Divorced	Unfit to work	Less than £400	30 per cent most deprived neighbourhood	Stroke, diabetes, obesity, epilepsy	Heavy smoker in the past, poor diet, high blood pressure, high blood cholesterol levels, diabetes, obesity, physical inactivity
William	57	Married/partner	Unfit to work	More than £800	10 per cent most deprived neighbourhood	Stroke, obesity	Family history of CVD, heavy smoker, poor diet, high blood pressure, high blood cholesterol levels, obesity, physical inactivity
Liam	76	Married/partner	Retired	Between £400—£800	20 per cent most deprived neighbourhood	TIA, diabetes, obesity, arthritis, spondylosis	Poor diet, high blood pressure, high blood cholesterol levels, diabetes, obesity, physical inactivity
Andrew	54	Married/partner	Unemployed with benefits	More than £800	40 per cent least deprived neighbourhood	Stroke, epilepsy	None
Abigail	74	Married/partner	Retired	More than £800	10 per cent most deprived neighbourhood	Stroke, thrombosis	High blood pressure
Jack	63	Single	Unemployed	Between £400—£800	10 per cent most deprived neighbourhood	Stroke, cancer	Smoker, poor diet, high blood pressure

a IMD 2015: Index of Multiple Deprivation (2015) is an official measure of relative deprivation for small areas in England.

### Data analysis

Thematic analysis was used as a tool of data analysis to understand and reflect people’s everyday reality (Braun & Clarke, 2006, 2013). In this study, the step-by-step guide from Braun and Clarke (2006) was used in order to identify patterns across data. The principal researcher (SEQ) became familiar with the data by reading the field notes and transcribed interviews. This first stage was discussed with PC and MG, and some first ideas were noted down. Codes were produced from the data—interviews, demographic information, questionnaires and field notes—by the principal researcher (SEQ). Two authors (PC and MG) checked the codes created across all transcripts. Codes were reviewed, some were renamed or transferred to another code due to its similarity, and finally they were organized into potential themes. At this phase, a visual thematic “map” was built to sort the codes into themes. The coding process and development of themes were discussed and reviewed with the research team. An inductive or “bottom-up” approach was used, so data was coded without trying to fit it into previous themes acknowledged by other studies, or the researcher’s interests (Braun & Clarke, 2006). Bury’s theoretical framework (Bury, 1982, 1991) linked to health inequalities literature then informed the overall analysis. NVivo software facilitated the analysis process.

## Results

Four main themes were developed using thematic analysis: (1) chronic illness as a disruptive experience (2) struggling for money, (3) lifestyle and health risks, and (4) reflections on current inequalities. Theme 1 was framed by biographical disruption theory (Bury, 1982, 1991). Themes 2, 3 and 4 expand the experience of chronic illness to encompass economic, lifestyle and environmental factors within health inequalitites contexts.

### Theme 1: chronic illness as a disruptive experience

Our findings describe biographically disruptive experiences of chronic illness (Bury, 1982). However, the nature and extent of disruption varied among participants. Some stroke survivors reported that their life changed completely—“*before the stroke I had a future*” (Derek)—by interrupting future plans. Others described their chronic illness experiences as a “*slow deterioration*” (Diana), which gives the idea of continuity rather than a disruption (Faircloth et al., 2004). The majority of participants reported that they had suffered a stroke (n = 11) in addition to having other chronic health conditions, including obesity (n = 7), depression (n = 2), diabetes (n = 5), and cancer (n = 1). Stroke survivors had to deal with both co-morbidities and the effects of the stroke. They reported sight problems, seizures, swallowing problems, memory loss, communication problems, paralysis and chronic fatigue after their stroke. Some were experiencing crises, such as financial hardship and illness, at the time. Hence, the onset of another illness added to their pre-existing problems.

Once you’ve got arthritis, it’s never going to go away like. It’s not a good thing to hear like but that’s another worry on you. (Stuart)

Participants with arthritis recognized their chronic illness as painful. The experience of physical pain drew the attention of the participants to their bodies. Awareness of bodily stages facilitated their recall of all the body parts that were damaged or painful. Participants frequently described their limitations in activities as well as their inhability to work as a result of their physical impairment. Liam pointed out “*the most annoying thing is that I can’t do what I used to do*”. He engaged in sports, photography and music concerts but he was limited because of neck spondylosis and arthritis. Some participants also highlighted restricted social components of life as disruptive experiences leading to isolation.

In this study the “what is happening?” (Bury, 1982) went beyond bodily states. Participants not only drew attention to their body but also their minds. Some reported mental health problems such as depression or anxiety. Commonly, participants could not cope well with the new situation caused by their chronic illness, which led to anxiety and/or depression. Mental health problems were considered more difficult to address and had bigger impacts on health and lives. Participants felt left out and that their mental state was not understood—“*I can walk up and down stairs and they think that’s fine”* (James). Participants found it hard to deal with and improve their mental state, and so were unable to sort out issues and seek help on their own. Indeed, some participants pointed out the necessity of being guided through the day. Participants were also conscious of how their mental state affected their personal life.

Well, without beating about the bush, I am depressed, and I’m aware that the way I am maybe affects other people. I mean, I said to you I was living in Area A when I had the stroke and now living in Area B. I mean, when I had the stroke I was living with my partner and she asked me to leave, and I’m sure that it’s because I couldn’t cope with having had a stroke and I was and am depressed. (James)

A new personal identity was acquired related to participants’ chronic health condition, suggesting that their sense of self had changed. Chronic illness prompted a new identity, revealed by participants identifying themselves with their chronic illness. For instance, “*my name is William and I had a stroke*”. When asked to say something about himself and to describe a normal day for him, William replied with the following words (note the times that he repeated “*I had my stroke*”). William’s narrative also reveals the process of re-thinking his life by emphasizing the “before” and “after-now” (Bury, 1982).

Well before I had my stroke I was erm … I was always on the go. But then when I had my stroke ’cause all my right side is weak, I need to get around with my stick now. When I had my stroke I was, my blood pressure was 220/101 so when the ambulance come they said we have to take you to erm … when they think you might have a heart attack … (William)

Terry and Jack also had to re-examine the course of their life story when a stroke occurred at the age of 47 and 28 respectively. Both participants assumed that strokes only occur among elders and felt the event as unanticipated considering their young age (Pound et al., 1998). As Terry said, “*I was too young to have a stroke*”. They had to re-evaluate the meaning of chronic illness as related to the ageing process. Lastly, the participants in this sample illustrate a wide range of responses and adaptations to chronic illness (Bury, 1991). After their stroke, Robert and James “*couldn’t be bothered”* to do sports, for instance. It “*didn’t make sense*” for them anymore. On the other hand, participants like Andrew and Louis kept themselves busy by volunteering or fixing things at home. Even though they were partially paralysed, they showed a strong determination and motivation to keep improving. Their achievements, such as being able to articulate words and finally talk, getting over their depression and being able to walk, made them proud. Participants normalized the effects of their chronic illness by trying to recover the pre-illness self, as far as possible (Bury, 2001).

### Theme 2: struggling for money

Participants faced tough financial situations. Diana, living in a little detached bungalow, explained “*I don’t live nicely, I just live*”. Derek, living on a state pension, said “*I wouldn’t call it comfortable*”. He had no savings and felt financially “*borderline*”. Participants did not consider, for instance, their car as a luxury but as a necessity to provide physical and mental mobility, and make life less dull. Some had become accustomed to these circumstances. No participants were working, all receiving disability, sickness, or welfare benefits. Terry was a builder and “*earning good money and living comfortable*” before his stroke, and he “*went from that to benefits*”, struggling particularly at first until he learnt how to adjust. Some participants felt depressed and hopeless because of their inability to work. Robert struggled to meet his bills from £73/week benefits. Jacob had no money for nine weeks after his stroke. Both participants related unpleasant experiences during benefit claims assessments, which made them feel judged and stigmatized. Many participants described complicated and exhausting procedures involving complex paperwork, and court or medical evidence compounded by their ill-health.

And having to go and claim benefits, it’s not a very nice procedure. Because they don’t treat you very well. […] When you go and claim benefits now, some of the staff think that they are doing you a favour by giving you benefits even though I’ve worked for over 20 years and I’m only taking out what I’ve paid in, you know, with tax and national insurance. So it’s not like they’re giving me anything, but they just sometimes make you feel like a second-class citizen. Not very nice. (Jacob)

As a consequence of financial hardship, participants had to control and prioritize expenses, carefully and consciously selecting their choices. Priorities varied among participants. James, the only participant who had to pay for prescriptions, said that medicines and food were top of his list. By contrast, others’ priorities were bills, mortage, and “*a roof over the head*” (Stuart). The tough financial situation made it difficult to save or have spare money. As Diana said, “*a pound will help*”. The majority of the participants were living day to day, economizing whenever possible, and developing money-saving strategies. Diana, whose only entertainment was the television, had frequently phoned the company to try to get the price reduced. Stuart had to sell some of his belongings to friends or second-hand shops to financially survive. James’s desperate strategy is exemplified in an email that he sent to the researcher (SEQ) some days after the interview.

I didn’t say this the other day as it is slightly indelicate/embarrassing. After my stroke when I got home I only used one piece of toilet paper at a time. It was a pointless attempt to try and save money. (James)

Worrying constantly about money became a way of life or “*second nature*” (Derek) for most participants. The stress and worry produced by the threatening financial situation had harmful effects on participants’ chronic health condition. Derek and James admitted that money could make a difference mentally.

It’s a constant stress. Most of the people in there will tell you when you walk in the door, every day you’re looking for the white envelope or the brown envelope to call you in, so it’s a constant, it’s constantly on your mind. […] You later learn that things like stress, it’s the worst thing in the world, stress. That’s one of the things you’ve got to learn to control, and you can only control that with this [money]. (Derek)

These feelings were shared by those around them. Relatives worried about participants’ health, and checked on them often. Participants’ families and neighbours were concerned about their delicate health condition which, indirectly, might have a negative impact on them. In order to relieve participants’ worries, their families did the finances for them.

Support came from family, neighbours, friends, churches, associations, and foodbanks; and it could be both material things such as food, money, and furniture, and less tangible things such as emotional support, phone calls on their behalf, and decorating and gardening. While some participants appreciated the help and support, material things and money made others feel embarrassed. Stuart felt ashamed for “*being needy*”, affecting his sense of self-pride. Although he did not ask, church, friends, and neighbours often gave him meals.

But sort of like I hide away from them [church, friends, neighbours] in the end because I don’t want to feel as though like I’m some sort of like I don’t know, for want of a better word like some sort of basket case like, let’s all help Stuart type thing. (Stuart)

### Theme 3: lifestyle and health risks

Several participants talked of “*unhealthy habits*” and how these could harm their health; in particular, smoking, unhealthy eating and insufficient physical activity. After his third stroke, Terry was restricted to a wheelchair, limiting exercise, so he put on a lot of weight. He felt unhealthy and noticed breathing problems from smoking and excess weight. Participants were, however, aware of ways to improve their health. Whilst some expressed intentions or actions to change these unhealthy behaviours, others reported not knowing how to improve their lifestyle or not being ready to change.

To eat healthy and be a non-smoker is the ideal thing, but … […] I can’t see me, at 54 years old I can’t see me changing that much now. […] I think I’m set in my ways now. As long as I can stay healthy and clot less. (Robert)

However, many of the stroke survivors reported a healthy lifestyle before the event. They expressed anger, disappointment and surprise, and they could not understand why it had happened to them. James’s narrative reveals the “why me?” question (Bury, 1982).

I never thought I was at risk of having a stroke. I mean, it’s not like they can tell me to lose weight, stop smoking, stop drinking. I don’t drink, I don’t smoke … […] I won the wrong lottery. They can’t tell me why I had the stroke. (James)

Participants were aware that their diet had undesirable effects on their chronic health condition. Despite their willingness to change and make healthy choices, some said that they could not afford the recommended diet. Losing weight was a common concern among those who identified themselves as obese, and recognized that healthier eating could make a difference and improve diabetes, blood pressure, and mobility. Participants tried to eat healthily by controlling unhealthy food intake, that is, “*putting the brake*” (Diana) or “*cutting down*” (Clare). Nevertheless, the convenience of ready-prepared meals and the easily accessed take-away food were barriers to nutritious food choices. Diet was not always a matter of choice. On the one hand, participants had to rely on others’ cooking skills or decisions when living in a family or in a residential care home; on the other hand, they were prevented from cooking for themselves (by epilepsy, paralysis, or visual impairment). Diana and Andrew declared that their diet was better when they had got a carer, but it seemed to be frustrating for Andrew since he could not feed himself due to his paralysis.

Poverty influenced food choices. Buying the cheapest food and seeking special offers were frequent money-saving strategies, in addition to receiving food from family or friends. Although the participants were grateful and showed appreciation, they also felt embarrassed. Food banks also played an important role. Stuart and Jacob described the nice atmosphere and great treatment, but pointed out that “*people should never be in the position to use one*”. Jacob lost his job when he had his stroke. With no income for over two months, he was given a food voucher by the council. Although he found the food bank staff very welcoming and helpful, he found that going there was “*very degrading after working for so long*”. He felt embarrassed and went just once, and then not to the closest. Robert never went to a food bank because he was “*too proud to go*”. Experiences of hunger were expressed by the same participants. Jacob went hungry after his stroke. After his benefits finally came through he still panicked when “*the cupboards were empty*” four days before getting paid. Stuart had to carefully control his limited budget, resisting his desire for occasional take-aways. Buying the desired and required food previously taken for granted was not always straightforward anymore. Stuart described not getting many hot meals and missing some meals, necessitating strategies to feel full.

A lot of the time I do eat a lot of cereal to keep myself going rather than having meals. I will have cereal and a cup of tea and that, that’s what … […] You can get cereal really cheap and that so … […] I like the one from Aldi you can get that’s pretty cheap obviously, it’s the cheapest or what. I will eat anything, whatever I get given. But if I was going to buy I would just get the erm … it’s like the rice flakes with like bits of fruit in for 79p, Aldi’s own. […] Or try and get a cheap loaf and just try and get beans and toast and things. (Stuart)

### Theme 4: reflections on current inequalities

Participants expressed both dissatisfaction and contentment with the health and care systems. While some received welfare advice and rehabilitation support, others did not get any of these services. Access to care services seemed to be associated with the individual’s capability to seek them. Participants’ mental health problems were barriers to strategically managing their chronic illness and seeking out care services. Whilst the National Health Service (NHS) was theoretically there to provide help, participants often had to request or seek support to receive it, rather than it being offered.

Maybe the NHS, if we use the NHS, or the government, needs to be more proactive. Because when I had my stroke, I can’t be proactive. Maybe now I can be a little bit reactive, but then this is what I was saying, three and a half years ago I couldn’t even react. (James)

Information about accessing care was lacking. Derek complained that he did not get any help when seeking information about care services. Terry was confused and disorientated when asking about cholesterol results. Another frequent complaint was delays for GP appointments, surgical procedures or other healthcare services. Several participants characterized the government and NHS as institutions that were not helping them manage their illness. Derek believed that there were not enough social policies, and James voiced disappointment and scepticism about health policies, highlighting that although prevention can save money and is more desirable than cure, it is chronically underfunded.

Participants often asserted themselves, highlighting missing rights, and dissatisfaction about the way they felt within wider society. These reflections about wider socioeconomic contexts shaped and influenced their experiences of managing their chronic illness as individuals living in a developed country. The majority of participants lived within the most deprived areas of a city in North West England. Shops were scarce, and there were few green spaces or community centres. The North-South divide emerged from Stuart’s interview. He seemed to feel he was being treated as a second-class citizen or dismissed in comparison with people from the South. Some inequalities were identified.

It’s just the people who are running the country at the minute, they just don’t care. That is the whole problem with the country. […] The way I’m feeling it’s just like a class thing all the while like. That’s the whole problem with anything to do with this country from my opinion and my visions from where I come from in the North, it’s just a class divide, and everything is a class divide, down to sports, down to looking at people. People don’t live in the real world from the South and that, everything is taken for granted up there because they’ve got everything, jobs, everything all up there. All the money seems to go up there, but it’s not easy up North and that. (Stuart)

## Discussion

Our findings describe the everyday realities for people with CVD and other chronic illnesses living in economically deprived neighbourhoods and experiencing economic hardship. These experiences are discussed within their contexts of relative poverty and social disadvantage in a developed country, using biographical disruption as a theoretical framework (Bury, 1982, 1991).

This study extends the commonly described “life-changing event” paradigm (Bury, 1982), as the nature and extent of disruptions described were more complex. Participants’ responses and pathways to overcome the chronic illness differed, as did their coping strategies. Our 14 narratives reveal diverse experiences of chronic illness, reflecting the severity of sickness and disability, other personal or psychosocioeconomic circumstances, point in the life course, lived environment, and access to resources. The concept of “*slow deterioration*” indicates that the individual’s life was not disrupted by the onset of the chronic illness but rather the chronic illness was part of individual’s life story (Faircloth et al., 2004). Whether the chronic illness was a single event or one among a series of adverse events or circumstances also influenced how the biographical disruption was experienced or managed (Reeve et al., 2010; S. Williams, 2000a). The concurrence of more than one chronic illness was common among the participants, and their narratives shifted from one illness to another (Faircloth et al., 2004). Some of the individuals in this sample were already familiar with pain, financial hardship, and death; so the onset of (another) chronic illness was not experienced as unusual crisis (Pound et al., 1998). For Stuart, his arthritis was nothing unexpected. He seemed to add this health problem to his “life-list” since he accepted that his arthritis would not disappear and that there were other worries in his life. Moreover, the experience of chronic illness may be felt and faced differently in the individual´s biography depending on the type of chronic illness (e.g., cancer may be more disruptive, and arthritis more anticipated). Co-morbidity suggests that the concept of chronic illness as biographical disruption should be reviewed by including all illnesses that shape an individual’s experience of ill health.

Biographical disruption confines pain exclusively to bodily stages (Bury, 1982); nevertheless, in this study, participants not only acknowledged physical discomfort but also psychological pain, which impacted on their health and lives. Fragile mental states affected relationships and social interactions, leading to isolation or incomprehension. This has similarly been found among Danish stroke survivors who reported limiting their activities and participation outside home, showing avoidance behaviour (Pallesen, 2014). Mental health problems seemed difficult to tackle and assess for participants in this sample. Anxiety or depression made them mentally dependent on someone who could tell them what to do when they faced problems, since it was difficult and demanding to sort out relatively trivial issues on their own. Our participants described not seeking professional help for mental health problems in contrast to their help-seeking for physical symptoms and disabilities. Patients’ access to medical knowledge allows them to separate the disease and selfhood, which is key to facing the physical effects of the chronic illness and legitimating individuals’ actions (Bury, 1982). These physical features of the chronic illness were labelled as “now-visible disease” by Bury. However, for some participants in this study, the difficulty of self-managing the chronic illness was also psychological. For this reason, we propose to broaden Bury’s concepts of “now-visible disease” and “selfhood”; and add the term “non-visible disease” to the model. The term “non-visible disease” suggested here indicates the psychological features of chronic illness. Hence, individuals experiencing a disruptive event may re-examine the relationship between the “now-visible disease”, the “non-visible disease” and “selfhood” when facing the effects of chronic illness. Other authors have recommended going beyond bodily signs (Felde, 2011) and introducing emotional capital or cognitive elements to better understand chronic health narratives (Reeve et al., 2010; S. Williams, 2000b).

The key elements of biographical disruption theory (Bury, 1982) were found in this study. Lives and taken-for-granted features had to be reformulated following the chronic illness onset (Bury, 1982). Participants frequently described inabilities to do what they used to, for instance, play sports or work, narrated as the “before” and “after” chronic illness onset. Biographical disruption theory includes re-thinking personal biographies, something commonly described in our study. Stroke survivors developed a self-imposed personal identity with lives revolving around their stroke and its after-effects. Stroke Association membership might have contributed to this as our participants were located in a stroke-focused atmosphere that inclined their mental state towards the incident of their stroke and its consequences. Thus our recruitment methods and interview setting might have emphasized their stroke self-identity descriptions. Moreover, re-evaluation of personal biographies after a stroke when relatively young was also found in this sample. Some participants could not understand why this had happened to them, believing that only elders have strokes. Stroke survivors in London’s East End assumed that the event during old age was “inevitable” while having a stroke when young was worse, leading to feelings of anger (Pound et al., 1998). The final response to the disruption aims to face and normalize the everyday situation (Bury, 1982). For some of our participants their struggle in overcoming the disruption was notable. While some participants “*couldn’t be bothered”* to pursue previously enjoyable activities, others tried to recover their pre-illness self by volunteering or doing DIY projects, also described in earlierm studies (Bury, 2001; Hubbard et al., 2010).

The financial hardships described are placed within current “austerity” policies. Since UK welfare reforms were introduced in 2010, public spending has been considerably reduced (Barr et al., 2017). Wide-ranging benefits and tax-credit reforms as well as public health, NHS and social care changes have increased the health gap for disadvantaged areas (Whitehead, 2014). These budget cuts might have aggravated participants’ economic hardships, requiring adaptations to deprived ways of living. All their most expensive belongings, such as their home, car or television, were perceived as limited, being barely affordable within welfare benefits. Many expressed a sense that there was insufficient money to live decent lives during austerity, leading to feelings of deprivation, unhappiness, and sensed injustice at having worked hard until a disruptive experience with consequent financial adversity and negative health impacts. All our participants were receiving welfare benefits. The procedure of claiming and assessment for benefits is frequently described as disproportionate and frustrating, which may lead to suicidal feelings and embarrassment (Whittle et al., 2017). The local administrating institutions possibly potentiated this stigma and, consequently, participants might have felt branded as “shirkers” and “scroungers” (Kayleigh Garthwaite, 2011). In addition, the benefits claiming procedure is often misunderstood and hard to access for people with chronic health problems, due to the scarce information and complex claim forms (Chapple et al., 2004). Stroke survivors, for instance, might face mobility, communication, and cognition challenges, which make the procedure more arduous. This procedure is especially hard for those with debilitating mental health problems, triggering under-claiming (Frost-Gaskin et al., 2003). Financial hardship was accompanied by experiences of worry and stress, which had noticeable negative effects on participants’ health conditions. Relatives were also worried about participants’ health, and helped their financial situation by giving, for example, food, money, or furniture. While some participants appreciated the help, others felt embarrassed for being in need. This may have potentiated stigma and thus prevented participants from asking for more help.

Smoking, unhealthy eating, and insufficient physical activity were suggested as the main barriers for a healthy lifestyle by our participants. In addition, factors affecting food choice were also acknowledged by the participants. Similar findings were described in the UK National Diet and Nutrition Survey (2003–2007) in low-income populations (Nelson et al., 2007). Among factors affecting food decision-making, financial restrictions had a great impact on the participants, leading to unhealthy food selection and food poverty. Participants were aware of the negative impact of an inappropriate diet on their cardiovascular health but the recommended diet was often described as unaffordable. Further research suggested that consuming an unbalanced diet could have a detrimental health impact (Garthwaite et al., 2015; Laraia, 2013; Seligman & Schillinger, 2010; Wolfe et al., 2003). The conscious selection of the cheapest food and price-reduced meals was determined by available funds. When it was difficult to afford food, participants in this sample relied on family, friends, or food banks. Feelings of shame resulted in meal-skipping and poor nutrition, worsening participants’ physical and mental health. Participants reported worries about running out of food and hunger. These experiences were similarly reported among foodbank users in the UK (Garthwaite et al., 2015), and food insecure families living in South London (Harvey, 2016).

Whilst some participants were content with service providers, many were not, disapproving of waiting lists, insufficient information, and limitations of prevention policies. The “logic of managed choice” in the UK assumes patient’s accountability for their own health and resource utilization (Pilgrim et al., 2010). Various factors could prevent or encourage individuals to seek help and access healthcare. For instance, trust in the NHS encourages patients to seek healthcare as well as improving patient satisfaction and adherence to treatment, which indirectly influences health outcomes (Calnan & Rowe, 2005). On the other hand, perceived stigma may act as a barrier to accessing healthcare, worsening chronic illness management, and quality of life (Earnshaw & Quinn, 2012). Our findings suggest that for our participants, proactive engagement by providers may increase uptake of services.

The majority of participants in our sample lived within the most deprived neighbourhoods in the UK according the IMD 2015 (Department for Communities and Local Government, 2015), which might have had a direct impact on their health (Airey, 2003; Bolam et al., 2006; Popay et al., 2003). Health policies stressing responsibility on lifestyle choices or “downstream” interventions do not favour people living in deprived areas (Jepson et al., 2010; McGill et al., 2015). Available evidence indicates that “downstream” interventions should be replaced with “upstream” interventions tackling core social problems and empowering communities since the focus on lifestyle factors is ineffective and enlarges the health gap (Capewell & Capewell, 2018; Marmot et al., 2010; O. Williams & Gibson, 2018). “Upstream” interventions like housing policies or greater availability of nutritious food stores and sport amenities may encourage healthy and suitable places in which to live (Graham, 2009). Improvements in social and physical environments can support good health and narrow health inequalities (Pearce et al., 2015) and reduce geographical inequalities (Bambra et al., 2010; Dorling & Thomas, 2009). In this study, participants described negative representations of their surroundings linked to feelings of disadvantage and deprivation. The concept of “second-class citizen” and the North-South divide were acknowledged in one interview as a result of the well-established inequalities between regions such as life expectancy, mortality rates, employment, welfare receipt, educational attainment, and health outcomes (Bambra et al., 2014; National Institute for Health Research, 2017; Whitehead, 2014).

### Strenghts and limitations

We believe our combination of biographical disruption and health inequalities theories better explains the diversity of chronic illness experiences (micro-context) and socio-economic realities (macro-context) interactions. This study adds the concept of “non-visible disease” to indicate the psychological components of chronic illness which is a promising area for further model development.

Our study has some limitations. Twelve out of 14 participants were recruited via the Stroke Association. Therefore, the findings may not be applicable to other deprived groups not represented in our sample, including people with other CVD and more severe impairments, and those who do not join similar healthcare associations. Gendered experiences of chronic illness management within individuals living in a deprived area were not explored due to the small number of females in this sample. Further research using biographical disruption theory should explore gender differences.

### Implications of the study

This study evidences the need for reviewing some concepts of the biographical disruption theory. We found that co-morbidity and the psychological features of a long term condition shaped participants’ experiences of ill health. This study identified that the nature and extent of disruption varied among participants as well as their responses and pathways to overcome the chronic illness. Future research should develop “narrative analysis tools” to identify different narrative chronic illness patterns (Edwards & Gabbay, 2007; Reeve et al., 2010) in order to support health care professionals in assisting management, mental pain, and strategies to cope and restore the disruptive experience of chronic illness.

This study showed that the mental health of the chronically ill participants was compromised. Mental health problems shaped ability to seek help, and this suggests that people living in disadvantaged areas managing a chronic illness might be in a cycle where the change depends more on the actions taken by the NHS or policymakers than their own. Health care providers should actively engage in helping chronically ill individuals living in deprived areas. Further actions should support these individuals by helping and advising with finances, offering emotional assistance, providing regular medical checks, and informing, and advocating healthy choices. In addition, health policies stressing responsibility for lifestyle choices should be replaced with “upstream” interventions empowering communities and avoiding potential stigmatization, so the health gap may narrow across the UK.

### Conclusion

Biographical disruption theory linked with health inequalities evidence may be a powerful combination to explore how experiences of chronic illness are underpinned by economic, lifestyle, and environmental factors and how they impact on individuals’ health status. The varied nature of participants’ narratives about their chronic illness indicates that the experience of biographical disruption is context-specific (Faircloth et al., 2004; Felde, 2011; Hubbard et al., 2010; Pound et al., 1998; S. Williams, 2000a): dependent upon previous experiences of illness, financial hardship, support from community networks or family, mental health issues, and geographical disadvantage. Thus, the variety of contexts creates heterogeneity of experiences (S. Williams, 2000a). Much of the struggle is hidden, linked to poverty, debt, isolation, emotional distress and mental ill health. Future work on biographical disruption should encompass wider health inequalities literature including psychosocial and economic features of chronic illness.

## Data Availability

The data that support the findings of this study are available on request from the corresponding author. The data are not publicly available due to privacy or ethical restrictions.
